# Longevity effect of a polysaccharide from *Chlorophytum borivilianum* on *Caenorhabditis elegans* and *Saccharomyces cerevisiae*

**DOI:** 10.1371/journal.pone.0179813

**Published:** 2017-07-20

**Authors:** Steve Thomas Pannakal, Sibylle Jäger, Albert Duranton, Amit Tewari, Subarna Saha, Aneesha Radhakrishnan, Nita Roy, Jean François Kuntz, Soraya Fermas, Darryl James, Jane Mellor, Namita Misra, Lionel Breton

**Affiliations:** 1 L’Oreal Research & Innovation, Bangalore, India; 2 L'Oréal Research and Innovation, Aulnay-sous-Bois, France; 3 Institute of Bioinformatics, International Tech Park, Bangalore, India; 4 Biochemistry Department, University of Oxford, Oxford, United Kingdom; 5 Sibelius Limited, Oxford, United Kingdom; Inha University, REPUBLIC OF KOREA

## Abstract

The traditional Indian medicine, Ayurveda, provides insights and practical solutions towards a healthy life style. Rasayana is a branch of Ayurveda known for preserving and promoting health, enhancing the quality of life and delaying the aging process. In the traditional knowledge, the Rasayana herb, *Chlorophytum borivilianum* (*C*. *borivilanum*) is regarded as a general health promoting tonic that delays aging and increases lifespan, cognitive function and physical strength. Aging is a complex and multifactorial physiological phenomenon that manifests itself over a wide range of biological systems, tissues, and functions. Longevity is an obvious marker of physiological aging. Simple model systems such as the single-cell budding yeast *Saccharomyces cerevisiae* (*S*. *cerevisiae*) and the nematode, *Caenorhabditis elegans* (*C*. *elegans*) are widely used to study the aging process and longevity. Here, we show that a polysaccharide fraction obtained from *C*. *borivilianum* increases the lifespan of *S*. *cerevisiae* and *C*. *elegans*, using an automated screening platform (Chronoscreen^TM^). Chemical analysis of this extract revealed a low molecular weight polysaccharide of 1000 Da, predominantly comprising Glu1→6Glu linkage. This polysaccharide showed significant dose-dependent extension of the median lifespan of *S*. *cerevisiae* by up to 41% and of the median lifespan of *C*. *elegans* by up to 10%. Taking cue from these results and the traditionally described benefits of *Rasayana*s on skin rejuvenation, we tested *in vitro* the polysaccharide for potential skin benefits. In a keratinocyte culture, we observed that this polysaccharide increased cell proliferation significantly, and induced synthesis of hyaluronic acid (HA), a well-known extracellular matrix component. Furthermore, when added to culture medium of human reconstructed epidermis, we observed an enhanced production of epidermal markers, e.g. CD44 and HA that are otherwise diminished in aged skin. Together, these results suggest that in addition to life-span extension of *S*. *cerevisiae* and *C*. *elegans*, a polysaccharide from the *Rasayana* herb, *C*. *borivilianum* may have beneficial effects on skin aging parameters.

## Introduction

Complementary and alternative medicines such as the Indian traditional medicine Ayurveda, recommends several interventions for prevention of diseases and promotion of health that need to be scientifically substantiated. Rasayana, a special branch of Ayurveda, aims at providing rejuvenating regimen, such as dietary recipes, herbal and mineral supplements, and health-promoting lifestyle to enhance quality of life and delay the aging process [1]. Traditional literature describes Rasayanas as interventions that reverse naturally occurring senility and improve mental competence, increase immunity against diseases, provide vitality and luster to the body and increase longevity [2]. As Ayurveda, Rasayanas are supposed to act holistically on the body by (i) improving the process of digestion and metabolism (Agni), increasing bio-availability of nutrients, (ii) optimizing the micro-circulation of nutrients through the body channels (Srotas), and (iii) enhancing the nutritive value of the plasma that becomes re-generated (Rasadhatu) [3,4]. Natural products and herbal medicines are promising sources of new agents and interesting candidates for the development of complementary and alternative treatments to conventional drug regimens. Traditionally, the Rasayana herb of the Hindi name safed musli (or Musli), also known as *C*. *borivilianum* (Family: Liliaceae), is a rich source of oligo- and polysaccharides and is traditionally regarded as a therapy that delays aging, increases life span, improves cognitive function and boosts physical strength [2]. Recent research on natural products has been directed towards polysaccharide chemistry and polysaccharides of *Ganoderma atrum* have been reported to reduce age-related oxidative stress through increasing the activities of superoxide dismutase, catalase, and glutathione peroxidase in mice [5]. Robert et al., reported that polysaccharides produced by *Klebsiella pneumoniae* exhibited beneficial effects on skin water content and micro-relief, and stimulated cell renewal and glycosaminoglycan biosynthesis [6]. Polysaccharides-based plant extracts were shown reducing trans-epidermal water loss (TEWL), which suggests that the regular use of such compounds could protect the skin barrier function [5,6]. As *C*. *borivilanum* is rich in polysaccharides, we focused on the isolation of a polysaccharide extracted from the root and investigated its potential skin beneficial properties.

Aging is a complex physiological phenomenon that impairs a wide range of biological systems, tissues, and functions. Today, age-related health problems represent a growing socio-economic challenge for society [1]. Current aging research aims at gaining longevity in which life span extension in humans is accomplished with a concomitant increase in the quality of life. In the last decade, significant progress has been made using model organisms, especially the nematode worm *C*. *elegans*, in delineating the genetic and biochemical pathways that are involved in aging, for identifying strategies of beneficial intervention in humans [7–9].

Health span is defined as a period of time where the physiological life of an organism is maintained in good health. Importantly, many tissues in a multicellular organism age at different rates [10]. Longitudinal studies increased evidence that simply adopting an appropriate diet and lifestyle may slow down or even reverse chronic conditions like coronary atherosclerosis [11]. Skin, as an externally visible organ, naturally exhibits changes during aging. Altered skin morphology is influenced by both the intrinsic aging program as well as extrinsic environmental factors such as chronic sun-exposure (photo-aging). In humans, some of these changes include a gradual atrophy and thinning of the epidermal layer by up to 50% from 30 to 80 years [12].

Identification of long-lived mutants in *C*. *elegans* paved the road towards a research dealing with a healthy lifespan extension [13]. *C*. *elegans* is a useful model organism with many experimental advantages, e.g. short generation time, easy maintenance, transparent body, simple but specialized organs which makes it a powerful tool for genetic analysis [14]. Importantly, *C*. *elegans* shares many similarities with mammals and humans, including aging and functional senescence. Aging worms experience a decline in mobility [15], chemotaxis [16] and reproductive capacity [17], and become increasingly susceptible to lethal infections [18]. These features make *C*. *elegans* an interesting system for studying aging pathways and age-related diseases in mammals.

Another model organism widely used in aging research is the budding yeast *S*. *cerevisiae*. It has been shown that yeast cells have a finite replicative capacity, which is measurable by the number of their daughter cells. Moreover, in a chronological lifespan, the length of time a yeast cell can survive in a non-dividing state, has been established as an additional read-out. Importantly, it has been shown that nutrient restriction can increase replicative the chronological lifespan in yeast [19].

Today, numerous pathways and interventions have been discovered that can extend lifespan in model organisms, of which notably nutrient or dietary restriction and reduced insulin/IGF-1 signaling (IIS) are highly conserved in vertebrates [20]. The IIS is an evolutionarily conserved pathway that regulates life span across many species, one example being *Drosophila melanogaster* that, like *C*. *elegans*, seems to have a single insulin-like receptor (dInR) that, when mutated, extends the life span, through modulating some aging processes [8, 20]. Therefore, experimental data obtained on simple model organisms could prove highly informative when mammals are considered [21].

In order to investigate the claims made in Ayurveda on the Indian Rasayana herb and its property to counteract the aging process and increase longevity, we first tested the polysaccharide of *C*. *borivilanum* on an automated longevity platform (Chronoscreen^TM^), based on the simple models, *C*. *elegans* and the budding yeast. Additionally, we assessed in skin cell cultures and in a living human reconstructed epidermis if the same extract could improve parameters associated with aged skin.

## Materials and methods

### Plant material

*C*. *borivilianum* roots were obtained from Natural Remedies Private Limited, Bangalore, India and were correctly identified and authenticated as *C*. *borivilianum* (Liliaceae) by the taxonomist, Bangalore, India. Powdered roots were extracted as aqueous decoction as per Ayurvedic Pharmacopoeia of India, using distilled water (Ayurvedic Pharmacopoeia of India, 2001).

### Extraction of polysaccharides from the roots of *C*. *borivilanum*

Roots were grounded with household mixer (Butterfly Brand, fitted with a 1 HP motor). Extraction with de-mineralized hot water (1500mL) of the powdered roots (0.1Kg) of *C*. *borivilanum* (1:15 w/v) at 85°C for 6 h, afforded a thick straw colored solution. This solution was filtered using a muslin cloth and the solution was then concentrated at 45°C, under reduced pressure using a rotavapor. The resultant aqueous extract was centrifuged at 5,500g for 10 minutes and then filtered using a glass filter (GFD, G-3, Whatman filter paper). The *C*. *borivilanum* extract was sterilized by filtration (0.45 μM syringe-filter; Merck Millipore Ltd, Vikhroli (E), Mumbai, India) and stored at -20°C until use.

### Purification by precipitation

The clear supernatant was cooled at room temperature for 1h and chilled ethanol (4°C) was added slowly into the supernatant solution under slow and constant stirring. The resultant mixture was left undisturbed at 4°C for 24h, after which the mixture was centrifuged at 10,000g for 10 minutes. The pellet formed under centrifugation represented an enriched polysaccharide. The pellet was repeatedly washed sequentially with anhydrous ethanol (50 mL) and acetone (50mL) and further freeze-dried for 48h to obtain an amorphous white powder.

### Carbohydrate and protein analysis

The total carbohydrates were determined by the phenol-sulfuric acid assay with D-glucose as standard at 490 nm [22]. Determination of amino acid composition was performed before and after protein hydrolysis using a dedicated Amino Acid Analyzer (L-8800 Hitachi) according to the methods by Guo et al [23]. The Hitachi L-8800 analyzer utilizes a lithium citrate buffer system and is optimized for physiological samples. The analyzer uses an ion-exchange chromatography to separate amino acids followed by a “post-column” ninhydrin reaction detection system. This can quantify individual amino acids down to the picomole level (~100 pmol). The hydrolysis procedure is ensured by 6N HCl for 24 hrs at 110°C. After completion of hydrolysis, HCl in excess was removed and the tubes were vacuum-dried for 90 minutes.

### Assay of total amino acids (colorimetric method)

The total amino acid content of protein free supernatants was estimated by a modified dinitrophenyl (DNP) derivatization method [24]. In brief, the polysaccharide (100 μl) or the reference standard (equimolar mixture of glutamate:glycine) was made up to 250 μl with 80% (v/v) methanol. Equal volume (250 μl) of borate buffer (100 mM borate, 150 mM NaCl, pH 9) was added to each tube, followed by 0.5 ml of 1-fluoro-2,4-dinitrobenzene, commonly called Sanger's reagent or DNFB reagent. Tubes were incubated at 45°C for 30 minutes and allowed to attain room temperature. After adding 1 ml of 0.25 M HCl to each tube and mixing, the optical absorption at 420nm was measured. Apart from reagent blank and reference standards, some of the samples also contained a known amount of added glutamate:glycine mixture for assessing the recovery of total amino acids through the different steps of assay procedure.

### Water content

Water content was measured by the Karl-Fisher titration technique using the universally followed method by introducing the sample directly in the methanol-based working medium, modified or not by adding formamide (1:3, v/v) to increase the sample solubility [25].

### Mass spectroscopy

#### MALDI-TOF analysis

The MALDI analysis was performed on the MALDI-TOF-TOF AUTOFLEX Speed mass spectrometer (Bruker–Franzen Analytik GmbH, Bremen, Germany) in the positive ion detection using the linear and reflectron detectors with a laser power value of 75 as defined in the Flex Control software of the instrument. The mass ranges using the linear and reflectron detectors were [800‐20000] and [400‐8700] Da, respectively. The MALDI-TOF instrument was calibrated with a peptide mixture. Several data were obtained including the Mw values and the general formula using the Polytools software and the smart formula application. In addition, MS\MS fragmentations were carried out using the LIFT and the CID modes. For the sample preparation, 1mg of the *C*. *borivilanum* polysaccharide and 15 mg of recrystallized 2,5-dihydroxybenzoic acid (DHB) matrix were prepared in 100μL and 1mL of water/acetonitrile (90:10, v:v), respectively. 2μL of the mixture of the extract solutions and the matrix with a volumetric ratio of 1 to 10 were deposited onto the Big Anchor Chip MALDI target using the conventional “dried droplet” method. To get a better co-crystallization, the deposits were quickly evaporated under air-flow.

#### HRMS experiments

ESI-HRMS analysis were performed by infusing the sample diluted in a water/acetonitrile mixture (90:10, v:v) into the ThermoFisher LTQ-FT mass spectrometer and scanning different mass ranges (covering intervals from 80 to 4000 Da).

### NMR spectroscopy

The NMR 1D and 2D spectra were recorded on a Bruker AVANCE 600 Fourier Transform spectrometer (Bruker BioSpin Inc., Fällanden, Switzerland), (frequencies: 600.18 MHz for ^1^H and 150.93 MHz for ^13^C) using a 5mm TCI cryoprobe, operating at 300K. The samples were analyzed at a concentration of 0.2% in heavy water (D_2_O), (Cambridge Isotope Laboratories, Inc., U.S.A.) without filtration.

### High performance anion exchange chromatography with pulsed amperometric detection (HPAEC-PAD)

Analyses were performed on a CarboPac PA1 anion exchange column (Dionex), using a ternary gradient Water/sodium hydroxide/sodium acetate, hyphenated with pulsed-amperometric detection.

### HPLC-CAD/MS

Analyses were performed on a Prevail Carbohydrate ES column (Grace), using a binary gradient acetonitrile/water, at a flow rate of 1.0 mL/min. Samples were 10μL injections of 10μg/mL each in 30/70 water/acetonitrile hyphenated with CAD and MS (ZQ 2000) detectors. This method allows oligosaccharide degree of polymerization (DP) to being estimated.

### Size exclusion chromatography (SEC)

Size exclusion chromatography combined with RI and MALLS (SEC) measurements were carried out using the multi-angle laser photometer combined with a P100 pump equipped with OH-Pak SB804 + 805 (Shodex, Munich, Germany) in series and differential refractive index detector (RI-150) at 25°C. The eluent was 0.1M sodium chloride (NaCl) + 300mg/L sodium nitrate (NaNO_3_) aqueous solution at a flow rate of 1.0 mL/min. Optical clarification of the solutions was achieved by filtering through 0.2 μm pore size filter (PTFE, Puradisc 13 mm Syringe Filters, Whatman, Kent, U.K.). Astra V software (Wyatt technologies, Santa Barbara, CA, USA) was utilized for data acquisition and analysis.

### Chronoscreen™ worm assay

#### Maintenance and strains

All assays were carried out using the GFP-tagged CB5586 *C*.*elegans* strain from *Caenorhabditis* Genetics Center (GCG, Caenorhabditis Genetics Center, University of Minnesota, Jackson Hall, Minneapolis, US). Worms were maintained on solid Nematode Growth Medium at 20°C seeded with E.coli OP50, as described in the Worm book (http://www.wormbook.org/chapters/www_strainmaintain/strainmaintain.html). FY631 (*MATα his4-917δ lys2-173R2*, *trp1Δ63*, *leu2Δ1 ura3-52*) is a strain from Fred Winston (Harvard) chosen for its realtively short lifespan to faciliate the aging studies.

#### Plate preparation

The lifespan assays were performed using the Chronoscreen™ platform, (Oxford, UK) and images taken using a Leica M205 FA fluorescence microscope (Leica Microscope, Wetzlar, Germany). *C*. *elegans* worms were grown by transferring chunks of agar from stock plates onto fresh 9 cm-diameter plates and incubating them at 20°C for four days. The 12-well lifespan analysis plates were filled with agar two days before bleaching. One day prior to bleaching, 25μl of autoclaved Elix® water was added to each well of the 12-well plate to help the spread of the previously prepared compounds. The compounds were diluted in vehicle (Elix® H_2_O) to required concentrations prior to treatment. Plates were left to dry off for 20 minutes under the laminar flow hood in sterile conditions followed by seeding (feeding) with fresh OP50 bacteria.

#### Worm synchronization

On the same day when the compounds were added to the plates, the eggs were prepared by washing each of the stock 9cm-diameter plates with 3 ml of M9 buffer. Eggs were then collected by performing 1x five minutes centrifugation at 3,000 rpm, bleaching them with hypochlorite solution and finally centrifuging them twice at 2,000 rpm to remove dead worms and bacteria. The eggs-containing M9 solution was then diluted to the appropriate concentration to reach an average of 8,000 eggs per ml of M9 buffer solution. As the viability of eggs after bleaching is around 75%, 25μl (equivalent to approx. 200 eggs) were dispensed to each well to achieve a yield of approx. 150 worms per well. Plates were then dried-off under a laminar flow hood for approximately 10 minutes. Plates were placed in the incubator at 20°C for three days to allow eggs to hatch and develop into L4 larvae. As soon as the worms reached this stage, a 50 μl of *E*.*coli* OP50 containing 30% of 10mg/ml FUDR was added to each well (“Screening Day 0”). The plates were then re-introduced in the incubator at 20°C for an additional day.

#### Imaging

The imaging was performed using a Leica M205 FA fluorescence microscope (ET-GFP filter set) and Leica Software LASX. Feeding with OP50 *E*.*coli* bacterial culture started from the day when FUDR was added (screening day 0), checked whenever the plates were imaged, and repeated as necessary.

#### Lifespan analysis

The lifespan analysis was performed using Sibelius proprietary software for processing imaging profiles into tabulated output of moving, live, and dead worms. Digital output was then analyzed by standard statistical package designed for worm and yeast survival analysis, the Oasis Survival Analysis Software (http://sbi.postech.ac.kr/oasis) [26]. The Log-Rank Test and the Fisher’s Exact Test were used to analyze data, draw graphs and obtain p-values.

#### Lifespan assay using *S*.*cerevisiae*

The assay for chronological lifespan in yeast requires growth of cells to stationary phase where cells stop cell division but remain metabolically active [27]. The yeast stains FY631 or BY4741 were stored in 50% YPD/50% glycerol at -80°C and an aliquot used to inoculate a starter culture in 5mls of YPD (http://www.yeastgenome.org/) and grown overnight at 30°C to OD_600_>1.0. For FY631, 10μl of culture were added to 1ml of synthetic complete medium (SCM) in a deep 96 well microtiter plate together with 1 μl of vehicle (A.R. H_2_O) or vehicle containing various concentrations of the *C*. *borivilianum* extract and grown with vigorous shaking at 30°C to maintain the cells in solution until stationary phase is reached. An O_2_ permeable membrane was used to prevent evaporation. For the strains derived from BY4741, 100μl of culture were added to 10ml of synthetic complete medium (SCM) in conical flasks together with 10μl of vehicle (A.R. H_2_O) or vehicle containing 10ng/ml of the *C*. *borivilianum* extract and grown with vigorous shaking at 30°C to maintain the cells in solution until stationary phase is reached. For both experiments, the cultures were maintained at 30°C with vigorous shaking for several days. Once in stationary phase, the number of viable cells in the culture was assessed at regular intervals using an out-growth assay in YPD on a Bioscreen C spectrophotometer (http://www.growthcurvesusa.com). The output data was transferred to YODA (http://yoda.sageweb.org) and survival curves calculated together with the area under the each curve and then analyzed using the Oasis Survival Analysis Software (http://sbi.postech.ac.kr/oasis). The Log-Rank Test and the Fisher’s Exact Test were used to compare the data, draw graphs and obtain p-values.

#### Ethics statement

Human dermal fibroblasts were isolated from mammary skin explants. Normal human skin was obtained from surgical residues of breast reduction surgery, with the patients’ written informed consent in accordance with the Helsinki Declaration and with Article L. 1243–4 of the French public Health Code. Patients’ written informed consents were collected and kept by the surgeon. The samples were anonymized before their reception by the authors. Only age, sex and anatomical site of samples were specified to the authors. The authors did not participate in sample collection. Given its special nature, surgical residue is subject to specific legislation included in the French Code of Public Health (anonymity, gratuity, sanitary/safety rules…). This legislation does not require prior authorization by an ethics committee for sampling or use of surgical waste.

Evaluation using human epidermal keratinocytes and dermal fibroblasts in monolayer culture Immortalized human epidermal keratinocyte cell line HaCaT (obtained from Professor Dr. N. Fusenig, Heidelberg, Germany as a kind gift) and human dermal fibroblasts (Caucasian) were used for the experiments as described previously [28]. Both cell types were maintained in Dulbecco’s modified minimal essential medium supplemented with non-essential amino acids and 10% fetal bovine serum (DMEM-FBS). Both keratinocytes and fibroblasts were maintained at 37°C in an atmosphere of 95% air and 5% CO_2_. Cells were sub-cultured by exposure to trypsin/Ethylenediamine tetraacetic acid (EDTA).The polysaccharide was dissolved in DMSO vehicle for the treatment and evaluated using 96 well plates. The proliferation was measured by quantification of DNA (CyQuant) after three days of incubation. The quantification of hyaluronic acid synthesis by HaCaT keratinocytes [29] was measured in the culture medium post five days incubation by ELISA [30].

#### Evaluation using human reconstructed epidermis model

EpiSkin™ reconstructed epidermis, cultured for 6 days on a collagen matrix at the air-liquid interface was supplied from Episkin SNC (Lyon, France). This model was used to evaluate epidermis markers (CD44 and HA) modulated during skin aging by ELISA, post polysaccharide treatment. Tissues were received at Day 6 and transferred into maintenance medium (2 ml/well) and incubated at 37°C, 5% CO_2_ incubator until the next day. Two batches of EpiSkin™ (Tissue 1 & Tissue 2) were used for polysaccharide treatments. Polysaccharide stock was prepared in DMSO (Sigma). The concentrations were 0.1, 1, 10 and 100μg/ml, diluted such that 0.1% DMSO concentration was maintained in the culture medium. Polysaccharide was added by systemic applications during 5 days, 2 times at Day 8 and Day 10 to the medium and cultures were incubated at 37°C, 5% CO_2_. Cytotoxicity at the tested Polysaccharide concentrations was evaluated by MTT test followed by microscopic examination of histological paraffin sections (H& E Staining) to confirm the tissue morphology. Among the 4 MTT tested concentrations, only three non-cytotoxic highest concentrations were evaluated for CD44 and HA ELISA assays. Cultures were harvested at Day 13 for protein extraction. After completion of treatment, skin inserts were rinsed with PBS. Two skin inserts of each concentration were pooled in 1mL of protein extraction buffer (Tris-HCl Buffer, triton, PMSF, protease & phosphatase inhibitor, Sigma) on ice for 20min, and centrifuged at 4700g for 15min at 0°C. Supernatant was centrifuged again at 16000g for 10min at refrigerated condition. Lysates were transferred in 96 deep well plates and stored at +4°C. Total proteins were measured using BCA kit (Thermo Fisher Scientific, absorbance at 562 nm). Specific ELISA kits for CD44 (Cluster of differentiation) and HA (Hyaluronic acid) were used from Antibodies-online and R&D, respectively.

#### Western blot

EpiSkin™ samples were treated with the polysaccharide at a concentration of 100μg/ml. DMSO treated skin model served as control. Polysaccharide was added twice at Day 3 and Day 5 and incubated at 37°C. Cultures were harvested at day 8 for protein extraction and Western blotting was carried out as per previous study [31,32].

## Results and discussions

### Carbohydrate analysis

The carbohydrate content of the extract reached 66.2% using the phenol-sulfuric acid assay with D-glucose as standard at 490 nm. Quantitative analysis showed that the content of free amino acids was around 2.5% and total amino acids by HPLC amounted to 3%, with a 2.9% water content.

### Mass spectroscopic analysis

Mass spectroscopy is a tool of choice, since it is selective and a powerful technique to obtain identification and structural information on compounds present in complex mixtures. As it only requires small sample amount, it is an excellent tool for understanding the composition of complex carbohydrates of plants. No single analytical method, alone, is capable of resolving carbohydrate structures, especially when present in complex mixtures. The key to the analysis of oligosaccharides is to find suitable MS fragments that are specific to a given structure and to allow alternatives to be excluded. Mass spectra provide ambiguous results when a product ion can originate from two or more precursor ions. Uncertainties in MS/MS spectra can often be removed by applying MS^n^ where product ions can be further examined and assigned to a specific structure.

#### MALDI-TOF MS analysis

**Fig 1:**
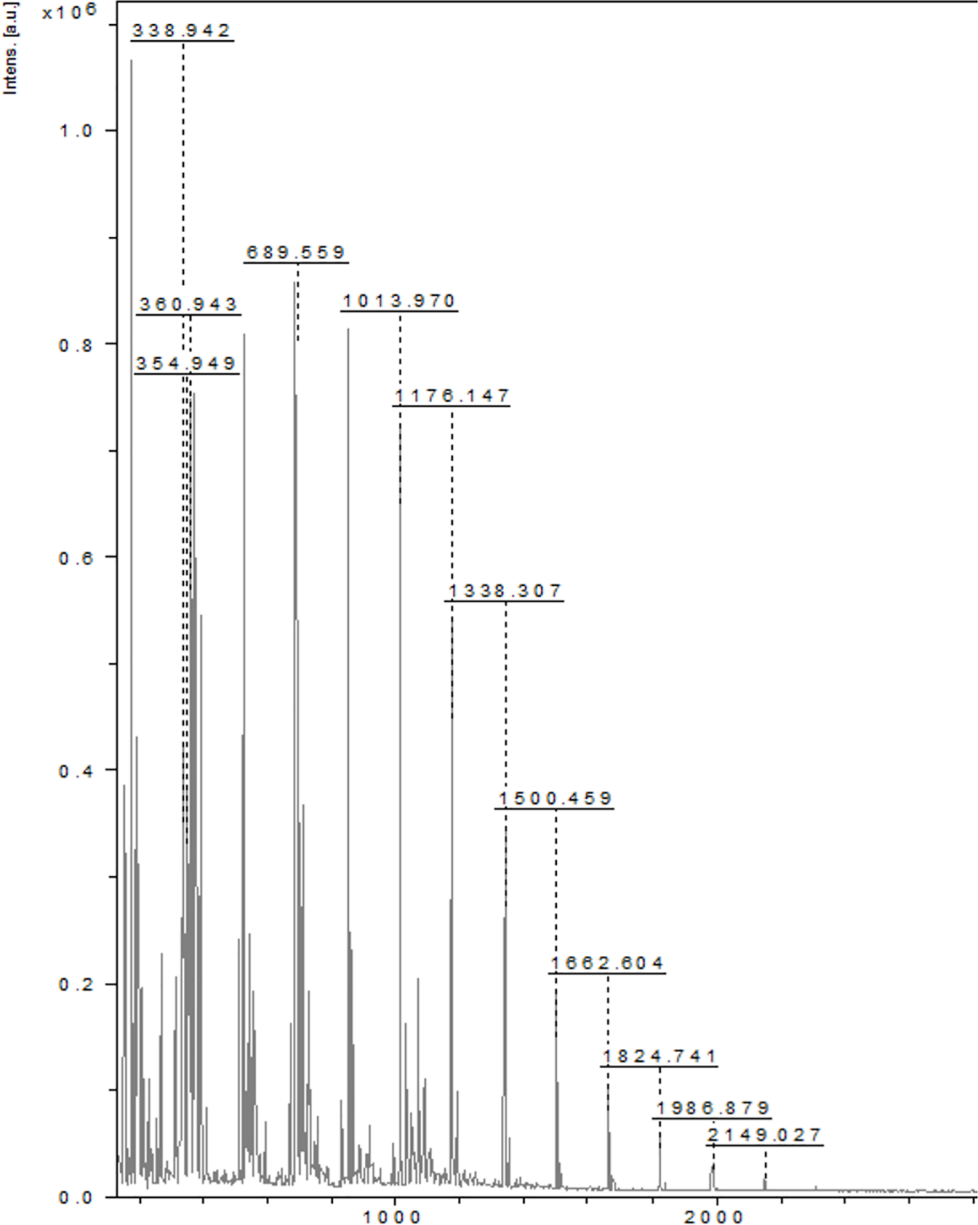
MALDI-TOF spectrum of polysaccharide. MALDI-TOF spectrum shows Gaussian ion distribution from m/z: 200 to 2200. The hexose species are detected in this sample until m/z 2149 (n = 13). The matrix used for the polysaccharide is 2,5-dihydroxybenzoic acid (DHB).

The MALDI-TOF MS showed the presence of *homo-*oligosaccharides with repeated glucose units with a mass, m/z 2149 Da (n = 13). This technique can be used to measure the molecular mass of carbohydrates up to 10^6^ Da, as shown by Mechref et al and Garozzo et al. on extracted and permethylated dextran standards [32, 33]. A successful characterization with MALDI-TOF MS provides the number-average molecular mass, the weight-average molecular mass, and the polydispersity index of the investigated macromolecules, as well as information concerning the end groups and the molar mass of the repeating units [33]. The coupling of MALDI-TOF MS with post-source decay (PSD) or collision-induced dissociation (CID) delivers more information on the structure of the parent ions and fragment peaks. During the last decade, the introduction of tandem mass spectrometry (MALDI-TOF MS/MS) overcame the limitations of the conventional coupled techniques such as PSD or CID [34]. In the instrument utilized for the present study, a LIFT^TM^ (CID mode) unit was integrated to gain this additional structural information. The unit is located in the flight tube, thereby offering a full-fragment ion spectrum within a single scan [35]. It has to be realized that the parent ion and resulting fragments are formed in the drift region of the MALDI-TOF MS/MS system. Different matrices suit different biomolecules and for carbohydrates the most frequently used matrix is 2,5-dihydroxybenzoic acid (DHB). Gaussian ion distribution from m/z 200 to 2200 is highlighted in “Fig 1”. Hexose species are detected in this sample until m/z 2149 (n = 13). Additional MS/MS experiments in the LIFT mode were performed using the precursor ions m/z 1666 and 1178. As mentioned above, ten series of fragment ions were determined in the MALDI-TOF CID spectrum. The molar mass difference between these series was 162.16 Da, i.e., the mass of one repeating unit of glucose. “S1 Fig” demonstrates a zoom of the molar mass range of 350–2,200 Da for the MALDI-TOF CID spectrum of the m/z 2,149 Da species, in order to identify each of the observed fragmentation series. They show consecutive losses of glucose units, which confirm the presence of mainly homo oligosaccharides.

#### SEC-RI analysis

The Size Exclusion Chromatography (SEC-RI) technique showed that the size of oligo/polysaccharide ranges MW 1,600 to 2,300. Many of the hydrophilic polymers are polyelectrolytes and, therefore, their elution properties in SEC is complicated by various non-exclusion effects, such as ion exclusion, polyelectrolyte expansion, molecular adsorption, and aggregate formation, which distort the normal SEC separation mechanism. These effects can be eliminated by increasing the ionic strength and changing the pH of the eluent so as to decrease the dissociation level of ionic groups both in the macromolecular chain and on the sorbent surface [35]. Physico-chemical properties such as structure, molecular weight and shape or conformation are primary factors controlling their functional properties. A typical molar mass sensitive detector is a multi-angle laser light scattering (MALLS). This detector has the advantage of providing structural information in addition to the estimation of molar masses. High performance size-exclusion chromatography (SEC-RI) of the polysaccharide shows one major peak “S2 Fig”. The results obtained in the SEC-RI are consistent with the HPLC-CAD mass spectroscopy described. The sizes of oligo/polysaccharide mixtures estimated through MALLS detection (dn/dc = 0.15 mL/g) showed Mn 1,600 (±9.1%) and Mw 2,300 (±15.5%) with Ip 1.5.

#### ESI/HRMS analysis

**Fig 2:**
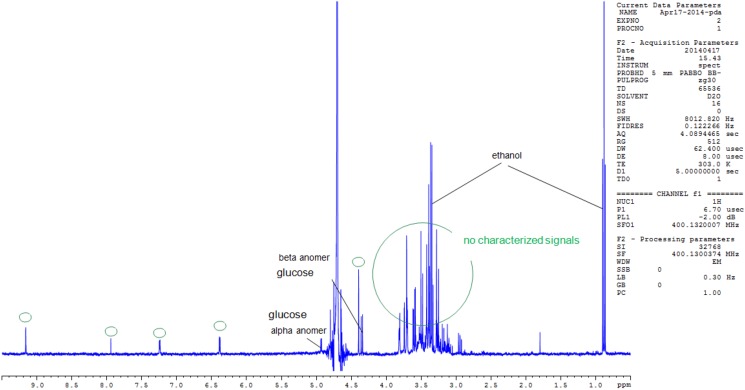
^1^H-NMR spectra of the polysaccharide recorded in D_2_O. Proton NMR of the polysaccharide displays characteristic signals between δ3.3 and δ5.6, typical of saccharides. The highly shielded signal at δ 5.5 is assignable to α-anomeric protons of glucose.

**Fig 3:**
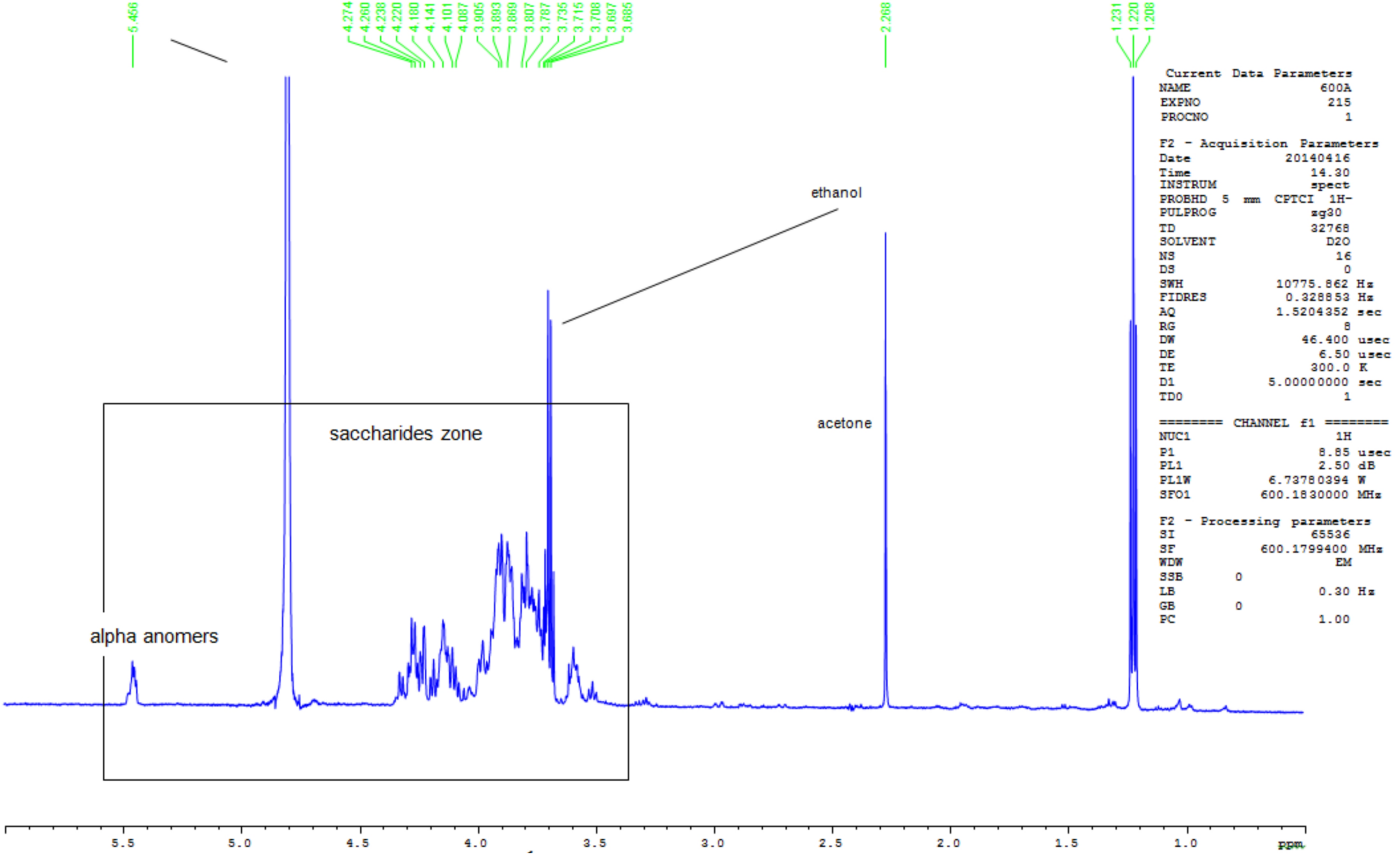
^1^H-NMR spectra of the polysaccharide recorded after acid hydrolysis in D_2_O. The NMR spectrum recorded after acid hydrolysis using 2% DCl for 1 hour at 100°C, shows characteristic signals of hexose carbohydrates. The spectrum clearly shows the nature of monomers and that the polysaccharide is composed of glucose.

The High-resolution mass spectrometry (HRMS) using ESI in the positive mode afforded an oligosaccharide of an intense peak at m/z 1,013. Additionally, a low intensity peak was observed at m/z 1089, corresponding to a compound of molecular formula C_51_H_86_O_23_, which can be attributed to the pseudo-molecular ion [M+Na]^+^ “S3 Fig”. This compound could be related to spirostanol saponin regarding its chemical formula and fragmentation pathway. The signals, around m/z 1337 on the spectrum, do not correspond to saccharides and are not characterized in this study. This is in agreement with the ^1^H NMR “Fig 2”, where the α-anomeric resonances are observed and the 2D DOSY MAP NMR spectrum of the oligosaccharides are shown in “Fig 3”. The ^1^H NMR spectrum of the polysaccharide after hydrolysis “Fig 2” shows a mixture of saccharides that differ from each other in the anomeric configuration (α,β- anomers of glucose) of the monosaccharide located at their reducing end.

#### NMR structural analysis

The 1D-^1^H-NMR spectrum showed mainly characteristic signals of saccharides (between 3.3 and 5.6ppm). The signal detected at 5.5ppm might be attributed to α-anomeric signals. In order to characterize the composition of the polysaccharides (i.e. the nature of monomers), an acid hydrolysis was performed under strong conditions (2% of DCl–during 1 hour at 100°C) and the NMR spectrum was recorded just after the hydrolysis. The NMR spectrum “Fig 3” shows characteristic signals of glucose and this information allowed us to infer that the polysaccharides were composed of glucose. Other signals in the high intensity regions were also detected.

#### 2D DOSY MAP analysis

**Fig 4:**
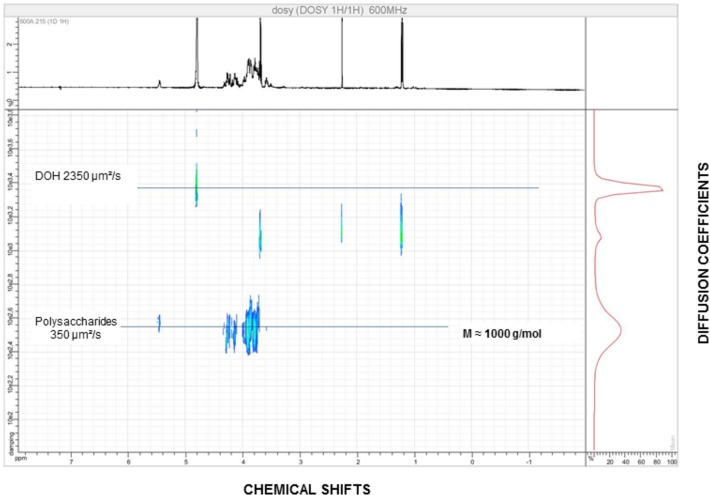
2D DOSY MAP of the polysaccharide recorded in D_2_O. The NMR DOSY experiment displays a low molecular weight oligosaccharide around 1000 g/mol. The x-axis corresponds to the chemical shifts (classical proton NMR) and the y-axis corresponds to the diffusion coefficients.

The NMR DOSY experiment for the polysaccharide was found containing an average molecular weight of around 1,000 Da. In the diffusion experiments (NMR DOSY), the different oligo/polysaccharides were separated by their diffusion coefficients. Theoretically, diffusion coefficient is used to directly correlate masses of the compounds, from which we obtain information on the mass of each polysaccharide populations. The 2D DOSY maps, “Fig 4”, are maps where the x-axis corresponds to the chemical shifts (classical proton NMR) and the y-axis corresponds to the diffusion coefficients. Important differences are observed between these 2D maps. The polysaccharide is mainly composed of oligosaccharides with a mass of around 1,000 g/mol.

#### HPAEC-PAD analysis

The High performance anion exchange chromatography (HPAEC) with pulsed amperometric detection (PAD) showed the extract to contain mono and oligosaccharides. Using this sensitive approach, the separation of oligosaccharides with a calculated molecular weight of ≈1kDa was possible “S4 Fig”. Inclusion of a 10 min column equilibration phase helped to analyze a complex mixture of low molecular weight polysaccharides within 60 min. Total saccharide composition was determined after 3h of acidic hydrolysis. The extract was mainly composed of 98% glucose and 2% mannose.

#### HPLC-CAD/MS analysis

**Fig 5:**
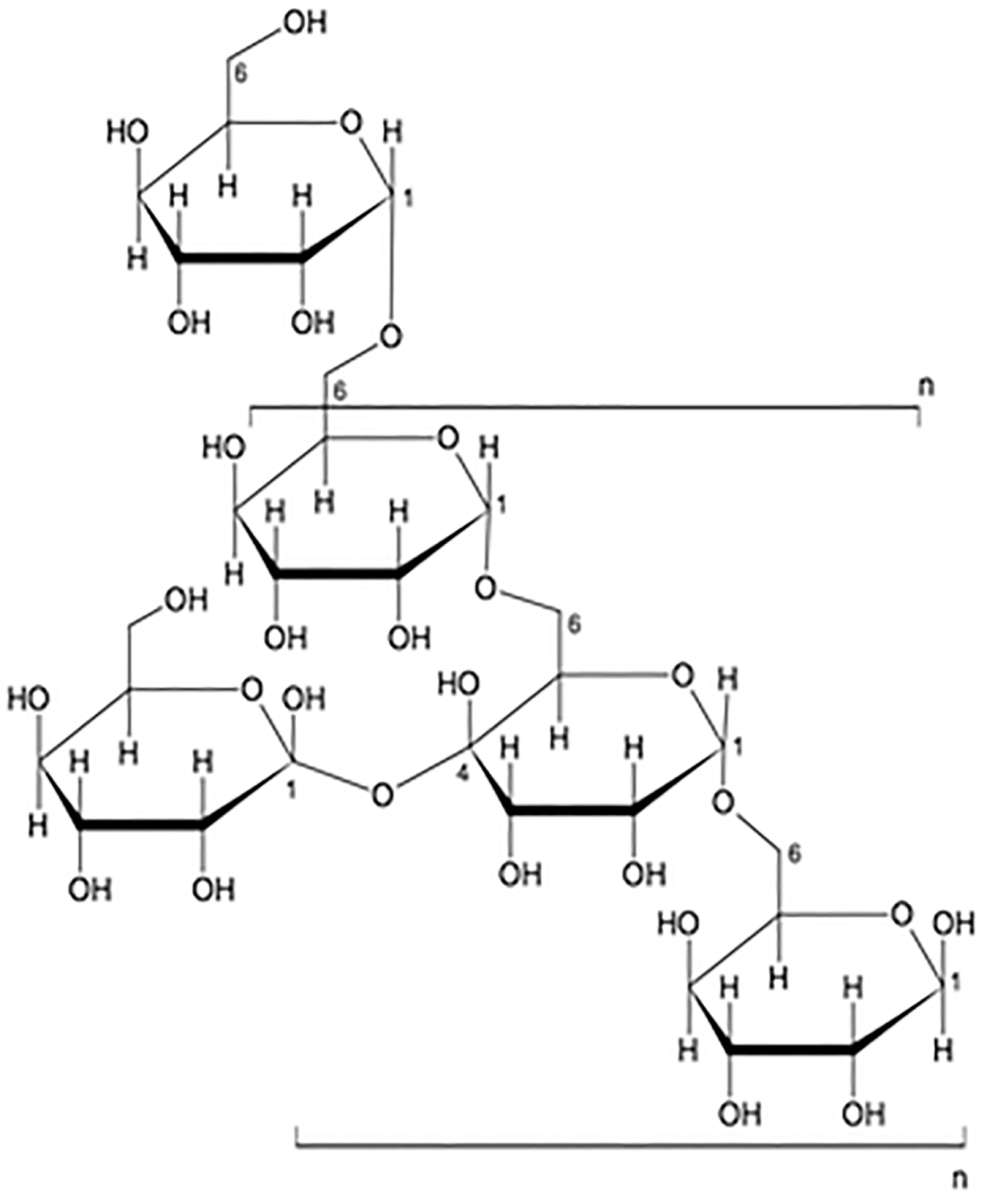
Possible repeating units of *C*. *borivilanum* glucans showing pre-dominantly Glu1→6Glu Linkage. Using mass spectroscopy technique coupled with a CID unit, the single peaks from the MALDI-TOF MS spectrum were fragmented. The sugar linkage was identified predominantly to be Glu1→6Glu, composed of glycans.

The LC-CAD-MS analysis showed the distribution of oligosaccharides with low molecular weight (Mw = 988 g/mol). Most glycan tandem mass spectra are produced by collisional induced dissociation (CAD), a technique in which selected precursor ions are dissociated by collision with gas atoms in a collision cell. Typically, the weakest bonds rupture to produce the most abundant product ions. It is possible to dissociate per-methylated glycans using high energy CAD that uses a MALDI TOF/TOF or ESI MS instrument [36], under which conditions bond rupture is kinetically controlled and cross-ring cleavage ions are more abundant for structural analysis. Charged Aerosol Detection (CAD) is a recently developed detector based on the detection of scattered particles, like in ELSD. The response obtained with the Corona CAD for nonvolatile analytes is less dependent on chemical structure than with other detectors. Charged aerosol detection response does not depend on analyte optical properties as with UV, or the ability to ionize, as with MS. These characteristics provide significant advantages for a wide range of quantitative methods. The polysaccharide analyzed using LC-CAD-MS shows the distribution of oligosaccharides with DP up to 20 and centered on DP5 (Mw = 988 g/mol) “S5 Fig”. Therefore, the final structure of the oligosaccharide from the comprehensive spectral analysis was predominantly composed of hexoses, and the linkage was identified as being Glu1→6Glu “Fig 5”. The approximate molecular weight was found to be 1000 Da.

#### Lifespan measurement in *S*. *cerevisiae*

**Fig 6:**
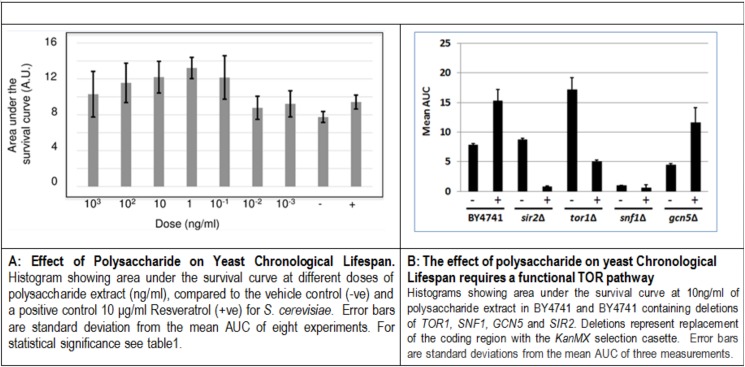
**A. Effect of polysaccharide on yeast chronological lifespan.** Histogram showing area under the survival curve at different doses of polysaccharide extract (ng/ml), compared to the vehicle control (-ve) and a positive control 10 μg/ml Resveratrol (+ve) for *S*. *cerevisiae*. Error bars are standard deviation from the mean AUC of eight experiments. For statistical significance see Table 1. **B: The effect of polysaccharide on yeast Chronological Lifespan requires a functional TOR pathway.** Histograms showing area under the survival curve at 10ng/ml of polysaccharide extract in BY4741 and BY4741 containing deletions of *TOR1*, *SNF1*, *GCN5* and *SIR2*. Deletions represent replacement of the coding region with the *KanMX* selection casette. Error bars are standard deviations from the mean AUC of three measurements.

The saccharide extract shows a dose dependent ability to significantly increase chronological lifespan, assessed as the area under the survival curve (AUC) compared to the vehicle control (Fig 6A). Optimal lifespan extension is observed at 1μg/ml. From the survival curves for the saccharide extract at 10μg/ml and 1μg/ml, mean lifespan is extended from 5.61±0.10 days (95% C.I. 5.42~5.79) for the vehicle control, to 7.48±0.07 days (95% C.I. 7.43~7.62) and 8.44±0.04 days (95% C.I. 8.37~8.51) respectively. The Fisher- Exact test confirms significant lifespan extension at 25% survival (*p* = 2.9 x10^-12^) and 50% survival (*p* = 3.1 x10^-12^) for the saccharide extract at 1μg/ml compared to the vehicle control. Thus, at optimal concentration, the saccharide extract leads to a statistically significant extension of mean lifespan by 34% and overall lifespan by 41%. To assess the nature of the conserved pathways known to influence lifespan and by which the polysaccharide extract from *C*. *borivilanium* extends lifespan, we used the BY4741 strain containing gene deletions in pathways known to influence lifespan. These included the pro-aging TORC1 pathway (*tor1Δ*) and the anti-aging AMPK pathway (*snf1*Δ) and deletion of two genes encoding either the Gcn5 protein lysine acetyltransferase (*gcn5Δ*) or Sir2, a sirtuin encoding a protein lysine deacetylase (*sir2Δ*), both of which have been implicated in aging pathways [37]. All four deletion strains have the expected influence on chronological lifespan (Fig 6B). If the polysaccharide extract is not dependent on a particular pathway or process, then an increase in lifespan will be observed. Gcn5 is NOT required for the polysaccharide extract to extend lifespan. By contrast, extension of chronological lifespan by the polysaccharide extract requires a functional TORC1 signaling pathway, as lifespan is reduced to levels similar to those in the untreated WT strain. Interestingly, the polysacharide extract further reduces lifespan in strains lacking either Sir2 or Snf1. This is likely to result from known genetic interactions and interdependencies between Sir2, Snf1 and the TORC1 pathways and suggests that the polysaccharide extract does not require these pathways for lifespan extension. We conclude that the polysaccharide extract may inhibit signaling by the TORC1 pathway to extend chronological lifespan in *S*.*cerevisiae*.

#### Lifespan measurement in *C*. *elegans*

**Fig 7:**
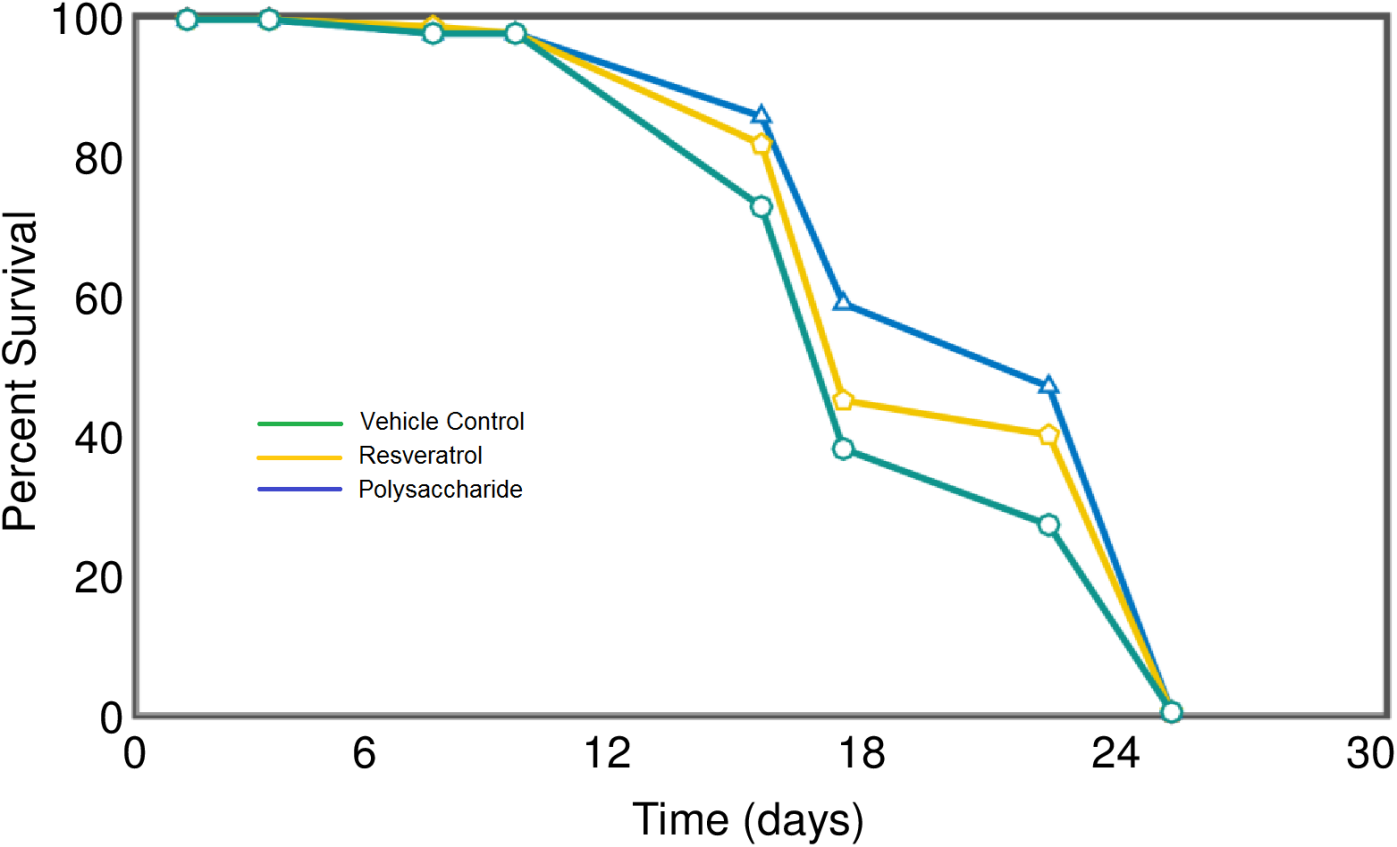
Effect of polysaccharide on *C*.*elegans* lifespan. Survival curves for *C*.*elegans* treated with 10μg/ml polysaccharide extract (blue line), 10μg/ml Resveratrol (yellow line) or vehicle control (green line).

The assay in *C*.*elegans* uses motility to monitor viability, which is then converted directly in live-dead digital profiles for statistical analysis of standard survival curves using the OASIS application [26]. The saccharide extract was tested at a range of concentrations from 10μg/ml to 1ng/ml and a significant lifespan extension observed for the highest concentration “Fig 7”. Treatment extended mean lifespan from 19.01±0.45 to 20.92±0.44 days with non-overlapping 95% confidence intervals (18.16~19.86 versus 20.04~21.80). Thus the saccharide extract leads to a significant 10% increase in mean lifespan (*p* = 0.0014) and a 22.7% increase in 50% survival (Fisher-Exact test, *p* = 0.0045). Resveratrol is a commonly used control compound for lifespan extension studies [38] (see Table 1). In the same experiment, resveratrol extends mean lifespan by 5% (p = 0.0484). Thus, the lifespan extending activity brought by the saccharide extract appears twice that of the positive control molecule, resveratrol.

**Table 1:** Effect of polysaccharide fraction treatment on median chronological lifespan and AUC in yeast.

S.no	Extract Conc.	Median Chronological lifespan		Area Under the curve	
	Polysaccharide fraction	ExtensionCompared toControl^a^	Effect of Treatment Relative to Effect of Resveratrol Treatment^b^	Change in AUC Relative to Control^c^	Effect of Treatment Relative to Effect of Resveratrol Treatment^d^
1	10μg/ml	No SignificantChange	No Significant Difference	No Significant Change	No Significant Difference
2	1μg/ml	+15%	No Significant Difference	+12%	No Significant Difference
3	100ng/ml	+37%	1.8 Fold Increase	+32%	1.4 Fold Increase
4	10ng/ml	+55%	2.7 Fold Increase	+40%	1.7 Fold Increase
5	1ng/ml	+23%	No Significant Difference	+26%	No Significant Difference
6	100pg/ml	No Significant Change	No Significant Difference	No Significant Change	No Significant Difference
7	10pg/ml	No Significant Change	No Significant Difference	No Significant Change	No Significant Difference
8	1pg/ml	No Significant Change	No Significant Difference	No Significant Change	No Significant Difference
9	Resveratrol 10μg/ml	+20%	NA	+24%	NA

^a^Effect of indicated treatment on Median Chronological Lifespan expressed relative to that of control treated cultures. “No Significant Change” indicates that the data generated for the indicated culture is indistinguishable from control treated cultures as defined by non-overlapping 95% confidence intervals.

^b^Effect of indicated treatment on Median Chronological Lifespan relative to the effect of Resveratrol treatment for those cultures for which treatment results in a significantly larger increase in Median Chronological Lifespan than observed for Resveratrol treated cultures (as defined by non-overlapping 95% confidence intervals). The size of this increase is expressed as a factor, relative to the effect of Resveratrol.

^C^Effect of indicated treatment on Area Under the Curve (AUC) expressed relative to that of control treated cultures. “No Significant Change” indicates that the data generated for the indicated culture is indistinguishable from control treated cultures as defined by non-overlapping 95% confidence intervals.

^d^ Effect of indicated treatment on AUC relative to the effect of Resveratrol treatment for those cultures for which treatment results in a significantly larger increase in AUC than observed for Resveratrol treated cultures (as defined by non-overlapping 95% confidence intervals). The size of this increase is expressed as a factor, relative to the effect of Resveratrol.

#### Effects on *in vitro* human skin models

**Fig 8:**
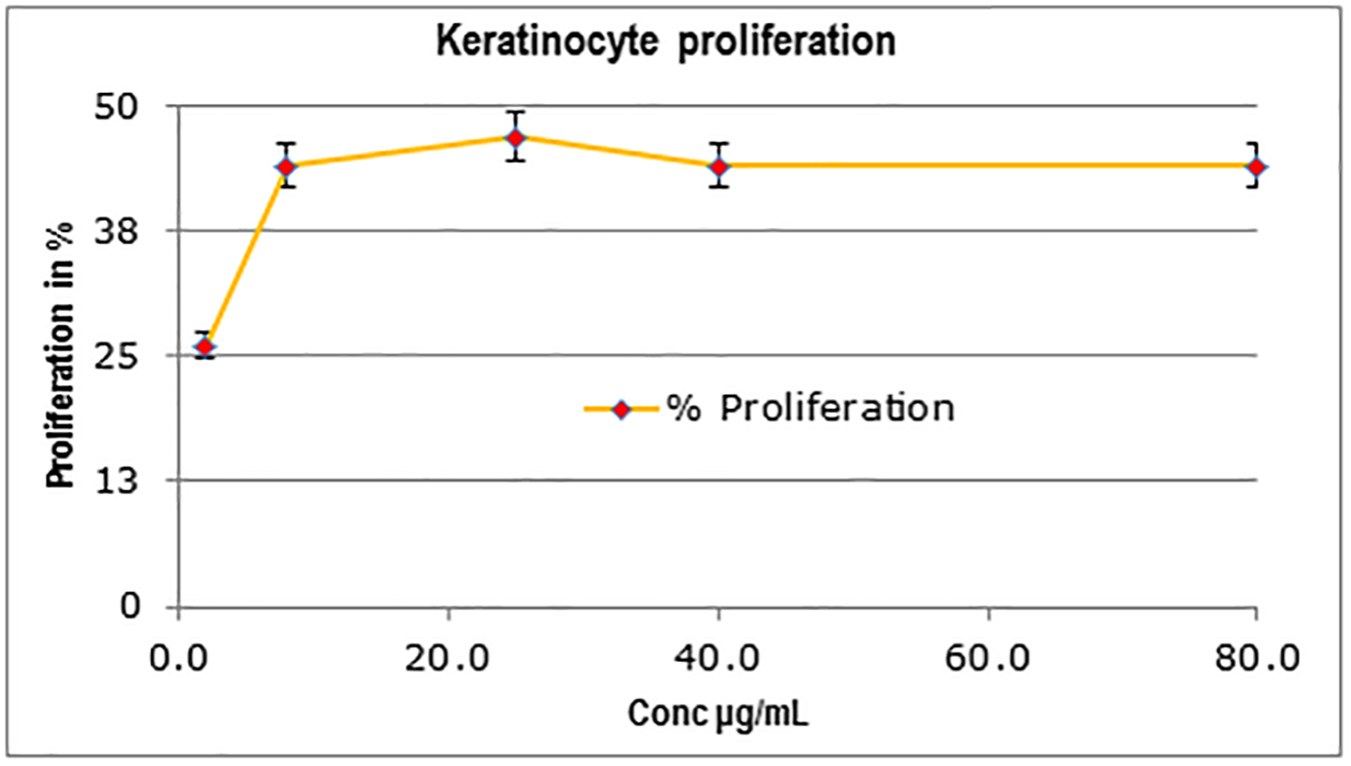
Effect of the polysaccharide treatment on keratinocyte proliferation. *C*. *borivilanium* polysaccharide displays 47% proliferation at 25μg/mL. HB-EGF, used as a positive control, displays 60% proliferation at 0.001μg/mL. *p < 0.05.

**Fig 9:**
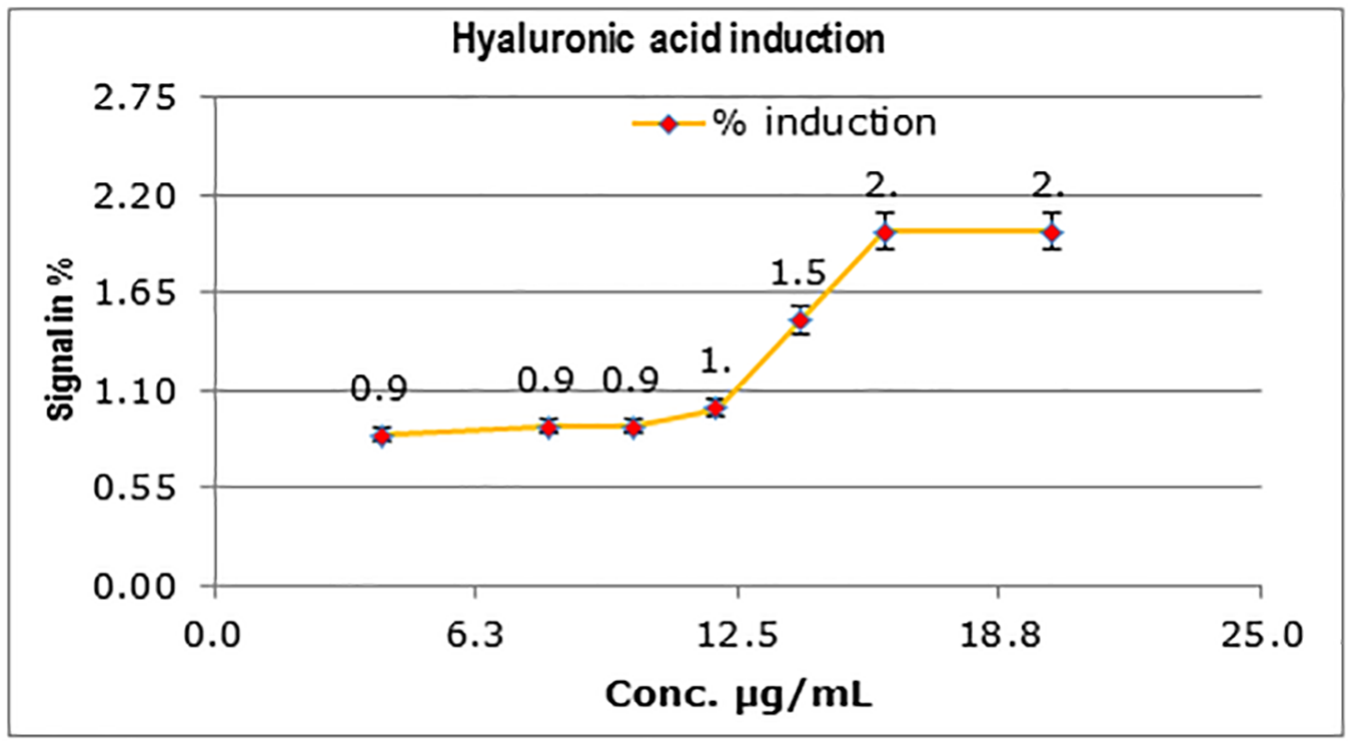
Effect of the polysaccharide treatment on hyaluronic acid synthesis in HaCaT keratinocytes. The induction of hyaluronic acid in HaCaT keratinocytes when *C*. *borivilanium* polysaccharide is added to culture medium. A two-fold induction of hyaluronic acid is observed with 16 μg/mL of *C*. *borivilanium* polysaccharide.

**Fig 10:**
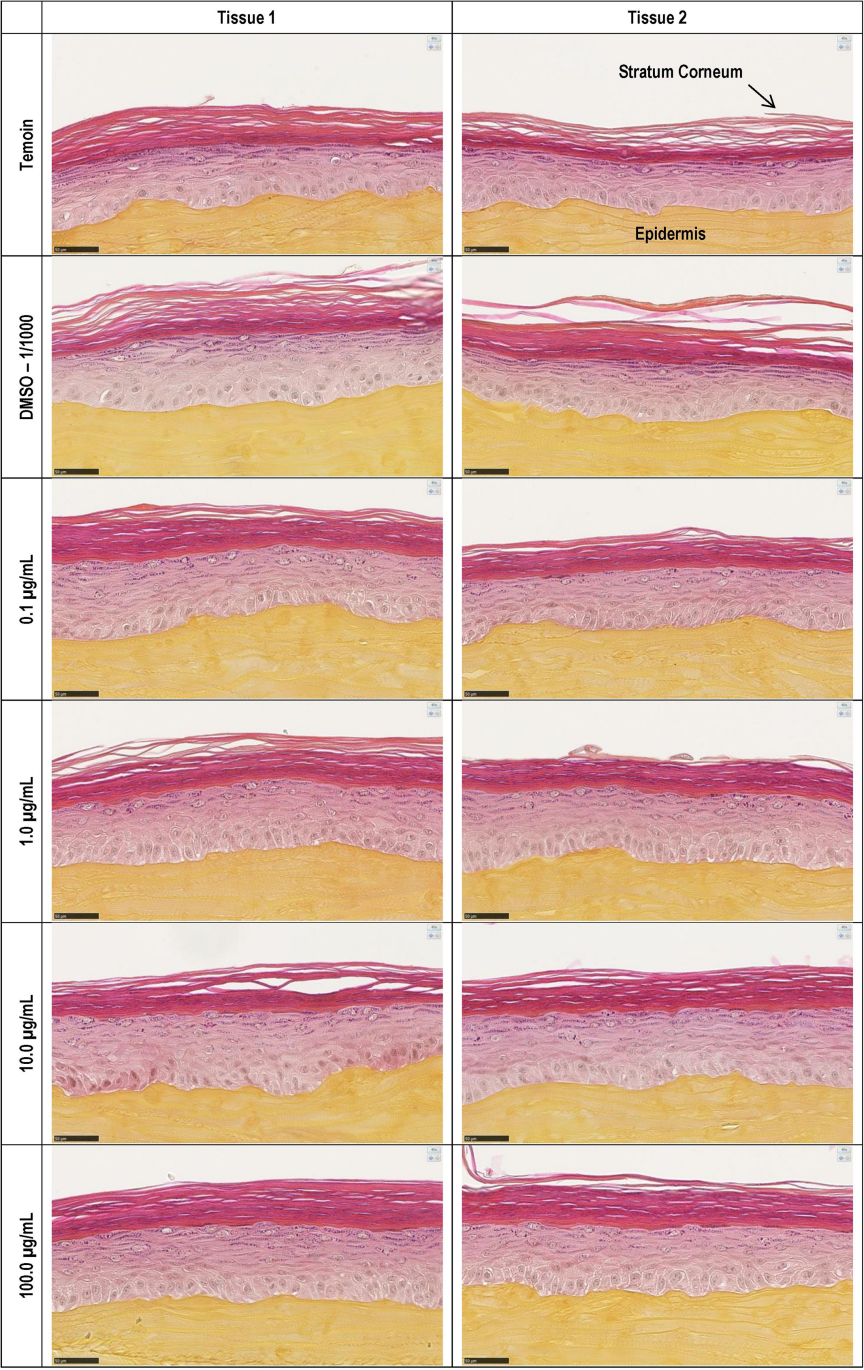
Histological analysis for effect of polysaccharide on tissue morphology. Microscopic examination of histological paraffin sections (H & E staining) showed standard and consistent morphology of reconstructed human epidermis (RHE). Thin and broad arrows indicate intact stratum corneum and Epidermis respectively.

**Fig 11:**
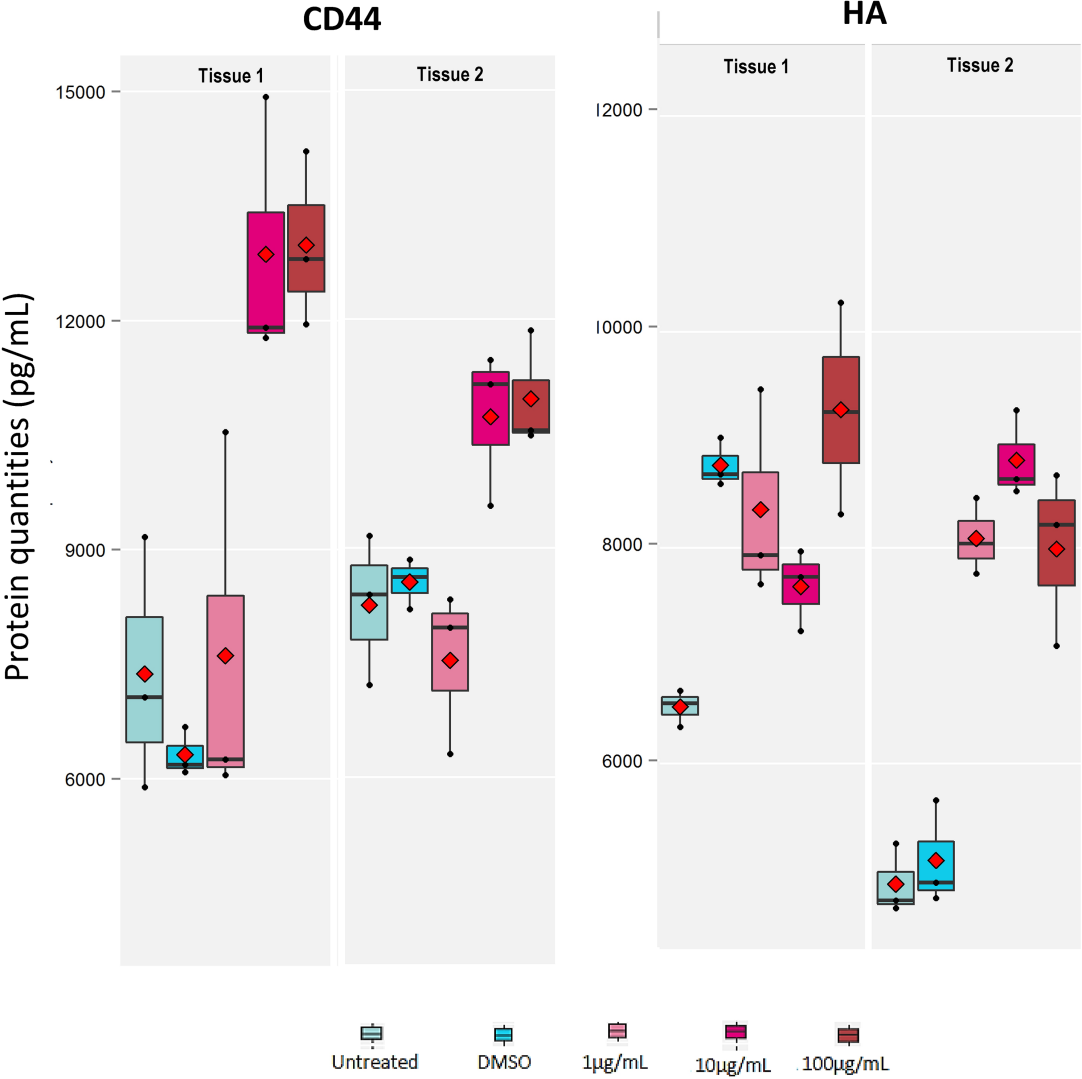
Expression of CD44 and HA in human reconstructed epidermis after treatment with polysaccharide. Proteins were quantified using ELISA in two separate EpiSkin^TM^ D6 tissue samples and at three different concentrations of polysaccharide (1, 10 and 100 μg/mL). Control was the tissue sample treated with solvent DMSO.

When added to culture medium, the polysaccharide showed significant dose-dependent increase of proliferation in HaCaT keratinocytes. At 25 μg/mL concentration of the polysaccharide, the proliferation was stimulated by 47% as compared to untreated HaCaT keratinocytes without any effect on cell cytotoxicity “Fig 8”. HB-EGF was used as a positive reference giving 60% proliferation at 0.001 μg/ml (data not shown). Further, the polysaccharide was tested in hyaluronic acid induction assay in HaCaT keratinocytes in a dose dependent manner. The polysaccharide increased significantly hyaluronic acid synthesis with an induction factor >4.8. A two-fold induction of hyaluronic acid was observed at a 16 μg/mL concentration “Fig 9”. The polysaccharide was also evaluated for its effect on two epidermal markers using ELISA on reconstructed human epidermis tissue samples EpiSkin™. Cell surface glycoprotein CD44 and HA are reported to be modulated during skin aging [39]. No cell cytotoxicity or negative effect on tissue morphology was observed up to 100μg/ml “Fig 10”. No cell cytotoxicity or negative effect on tissue morphology was observed at all the 4 concentrations of Polysaccharide tested up to 100μg/ml as determined by MTT assay (“S6 Fig”) and H & E staining respectively (“Fig 10”). The polysaccharide showed the strongest effect on CD44 protein induction at 100μg/ml (70%)Moderate induction of HA synthesis (31%) was observed at all three tested concentrations (1, 10 & 100 μg/ml) “Fig 11”. In addition, a 38% increase in the proliferation of human primary fibroblasts in culture was observed at 100μg/mL of the polysaccharide without any effect on cell cytotoxicity, while there was no effect on procollagen induction (data not shown).

**Fig 12:**
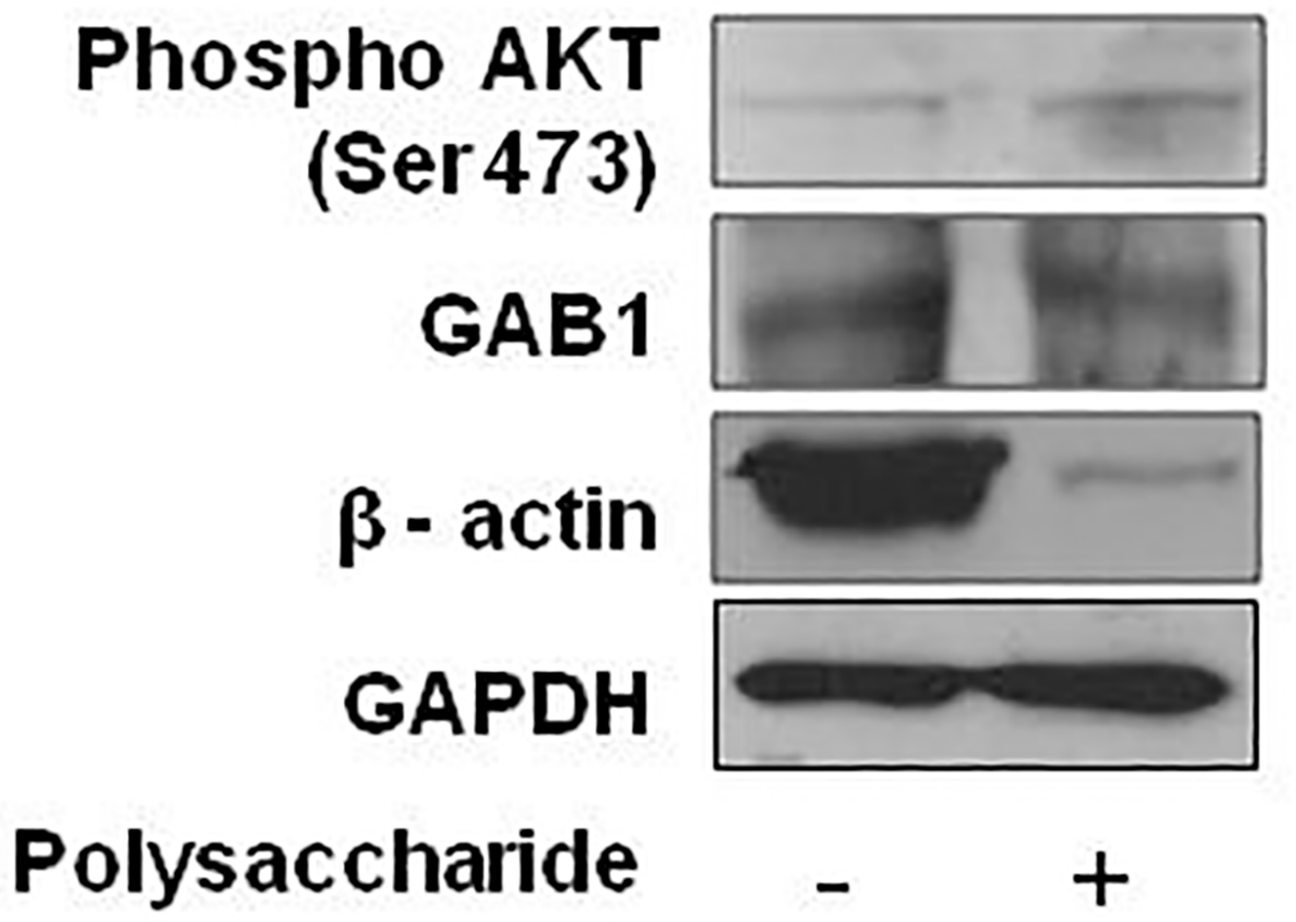
Western blot analysis of pAKT and GAB1 in human reconstructed epidermis after treatment with polysaccharide at a concentration of 100μg/ml.

HA/CD44 signaling has been implicated in keratinocyte cell growth and survival [40, 41]. We investigated the underlying mechanisms associated with beneficial effect induced by polysaccharide treatment using human reconstructed epidermis. We determined the levels of phospho AKT (Ser-473) using an *in vitro* model by Western blot analysis. As shown in “Fig 12”, treatment with polysaccharide resulted in the hyperphosphorylation of AKT at Serine 473 in human reconstructed skin model. In addition, total GAB1 (4) was found to be unchanged upon polysaccharide treatment. Studies have also shown that actin turnover regulates aging. In this study, we observed a decrease in the expression of actin levels upon polysaccharide treatment. This could be due to the role of actin in cytoskeleton remodelling. Taken together, our data indicates that the beneficial effect of the polysaccharide is mediated through the activation of PI3K/AKT pathway.

We established a method to isolate a rich water-soluble polysaccharide from the roots of *C*. *borivilanum* with an ideal yield. Through hot water extraction technique, we obtained a crude polysaccharide rich extract as a water-soluble light yellow color solution which could further be purified by cold ethanol precipitation to an amorphous powder with total yield of 30% and a high sugar concentration of 66%. We found that the polysaccharides were mainly composed of oligosaccharides with a mass around 1,000 g/mol. A detailed characterization of a defined polysaccharide sample was demonstrated using the MALDI-TOF MS technique coupled with a CID unit, which allowed single peaks from the MALDI-TOF MS spectrum to being fragmented. Using a combination of modern spectroscopic techniques, the approximate molecular weight was determined to be 1000 Da, predominantly the linkage was identified as being Glu1→6Glu “Fig 5”.

A range of herbal extracts have been shown to extend lifespan in *C*.*elegans* [36]. For example, it was shown that blueberry polyphenols [39], resveratrol from grape skin [40], the alkaloid Reserpine isolated from *Rauwolfia serpentina* and *Withania somnifera* root extract [41] extend *C*. *el*egans lifespan. The latter plant belongs to the category of ‘Rasayanas’, which are described as rejuvenators in the traditional Indian medicine. Therefore, Rasayana plants are considered as particularly promising candidates to investigate in longevity studies on model organisms. The characterization of potential anti-aging effects of a polysaccharide fraction from the roots of *C*. *borivilanum* in this study indicates that the polysaccharide fraction is biologically active and appears to up-regulate evolutionarily conserved networks controlling cellular anti-aging mechanisms in *S*. *cerevisiae* and in *C*. *elegans* [42]. Moreover, our in vitro experiments in skin cells revealed potential skin anti-aging benefits.

The Chronoscreen^TM^ technology, as a phenotypic high throughput screen, allows the statistically unbiased assessment of the anti-aging biological activity associated with cellular chronological lifespan. Using this novel automated high-throughput longevity platform (Chronoscreen^TM^), we showed that the polysaccharide fraction significantly extends the chronological lifespan of yeast by up to 41% (1μg/ml) in a dose-dependent manner, and the mean lifespan of *C*. *elegans* by 10% (10μg/ml). Furthermore, we have shown that the increase in yeast chronological lifespan requires a functional TOR pathway. Compounds previously identified as biologically active on the Chronoscreen^TM^ platform, have shown to have a high efficacy in reversal of senescence in human and rodent cells, reduction of toxic protein aggregates in yeast and worms, and increase in neuro-regeneration in rats (Chronos Therapeutics unpublished results). Similar to the polysaccharide extract used here, bioactive compounds generally increase lifespan between 5 and 50%. Larger increases in lifespan are generally accompanied by compromised growth or fecundity. These increases may appear small but large numbers for these experiments, made possible using the Chronoscreen^TM^, allow the robust detection of these effects. For example, resveratrol, well documented to improve healthy lifespan in many organisms used as a positive control on the Chronoscreen^TM^, produces a mean lifespan extension of around 5% in *C*.*elegans* and around 20% in yeast [36, 38, 43]. Interestingly, resveratrol has also been suggested to have skin anti-aging benefits [44, 45]. The longevity effect of *C*. *borivilanum* might be due to its specific polysaccharide composition. Earlier, Zhang Yusi et al., showed that a polysaccharide from *Bletilla striata*, traditionally used as an anti-aging herb in traditional Chinese medicine, increased lifespan in *C*. *elegans via* the insulin/IGF signaling pathway [46].

Aged human skin displays reduced epidermal thickness, flattening of the dermal-epidermal junction, decreased keratinocyte proliferation and loss of skin moisture [47, 48]. HA, a component of the extracellular matrix synthesized by fibroblasts and keratinocytes, plays a very important role in retaining water in the skin. In the epidermis, HA is found abundantly in the spaces between the skin cells. During skin aging, decreased epidermal HA has been reported [48]. Significant dose-dependent increase in cell proliferation and HA induction was observed in HaCaT keratinocytes treated with the polysaccharide. Epidermal markers are widely known to be modulated during skin aging, CD44, a glycoprotein that resides on the surface of the keratinocytes, is known to bind HA [49, 50]. The CD44 protein is a class I transmembrane glycoprotein with multiple roles in cellular processes including cell adhesion, growth, differentiation and motility [51]. It has an extracellular domain (ECD), a single pass transmembrane domain and an intracellular domain (ICD) [52]. CD44 undergoes extensive alternative splicing, especially in its ECD and has multiple isoforms expressed in different cell types [51]. CD44 functions as the major cellular adhesion molecule for hyaluronic acid (HA) and has a HA-binding site in its ECD [53]. Cleavage of ECD of CD44 is mediated by matrix metalloproteinases such as MMP9 [54] which is triggered by various mechanisms including the activation of Rho family small GTPases [52]. Cleaved ectodomains of CD44 can be of multiple sizes including 55 kDa and 25 kDa, depending on the isoform of CD44 being cleaved [54, 55]. The cleaved, soluble form of CD44 is known to have a greater binding efficiency to HA [56].

Once HA binds to CD44, it activates the secretion and activity of MMP9 [57] which in turn cleaves CD44, releasing the ECD or soluble CD44 fraction into the extracellular matrix [54]. This activates presilin/ secretase in the cytosol which cleaves the ICD of CD44 and triggers downstream signaling [52]. These biological endpoints were confirmed using reconstructed human epidermis. The polysaccharide induced CD44 protein expression with a moderate to strong effect and HA production with a moderate effect. Immunoblotting showed a significant decrease in CD44 levels (55kDa band corresponding to cleaved ECD of CD44) in polysaccharide treated reconstructed human epidermis (data not shown). This indicates an increase in HA-CD44 signaling that results in increased MMP9 activity. An increased activity of matrix metalloproteinases is associated with an increase in cellular migration. However, investigating the increase in cellular migration using reconstructed human epidermis is beyond the scope of this study. Our results indicate an increase in HA-CD44 signaling through the phospho-Akt signaling pathway in reconstructed human epidermis treated with the polysaccharide. Studies have indicated a strong association between HA-CD44 leading to PI3K-AKT activation through GAB1 (4).

Our data indicates that the beneficial effect is mediated through activation of PI3K-AKT pathway with a decreased expression of β-actin in response to treatment with the polysaccharide. Studies have shown that actin turnover regulates increase in ROS [58]. There is enough evidence linking increased ROS and skin aging.

Stimulation of keratinocyte proliferation may improve epidermal renewal and counteract the decrease in epidermal thickness during skin aging. The effects of the polysaccharide on cultured keratinocytes and in the reconstructed human epidermis model when added to culture medium resemble to those associated with biologically active retinols that have proven ability in the skin repair process [59]. Therefore, the *C*. *borivilanum* polysaccharide extract might be a beneficial ingredient for skin care products. In conclusion, our work on a polysaccharide from *C*. *borivilanum* in the model organisms S. *cerevisae* and *C*. *elegans* substantiated its age delaying activity described in traditional Indian medicine. In addition, our *in vitro* data on skin cells and human reconstructed epidermis suggests that *C*. *borivilanum* might also have some benefits on aged skin.

## Supporting information

S1 Fig MALDI MS/MS spectra of the m/z ions 1666 and 1178 for the polysaccharide. (TIF)

S2 Fig Size exclusion chromatography (SEC) chromatogram of the polysaccharide. (TIF)

S3 Fig ESI/HRMS spectra of the polysaccharide recorded in positive ion mode. (TIF)

S4 Fig HPAEC-PAD of the polysaccharide. (TIF)

S5 Fig HPLC-CAD/MS of the polysaccharide. (TIF)

S6 Fig Evaluation of cytotoxicity on reconstructed human epidermis (RHE) by MTT assay. (TIF)
