# Supplementary figures and images for: Longevity effect of a polysaccharide from Chlorophytum borivilianum on Caenorhabditis elegans and Saccharomyces cerevisiae
Source: PLoS One. 2017 Jul 20;12(7):e0179813. doi: 10.1371/journal.pone.0179813 (PMC5519035; doi:10.1371/journal.pone.0179813)

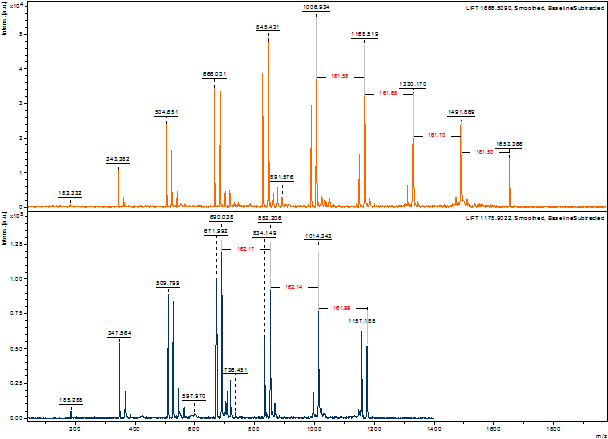

Supplement: S1 Fig — (TIF) [file pone.0179813.s001.tif]

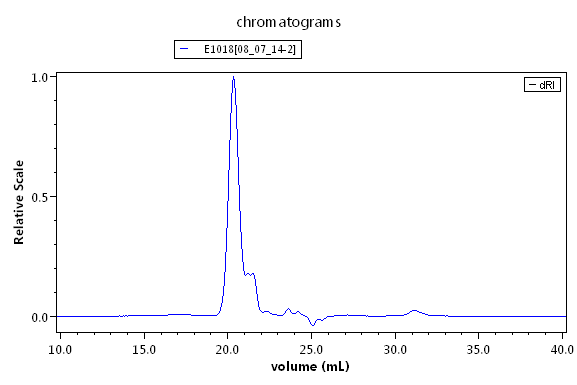

Supplement: S2 Fig — (TIF) [file pone.0179813.s002.tif]

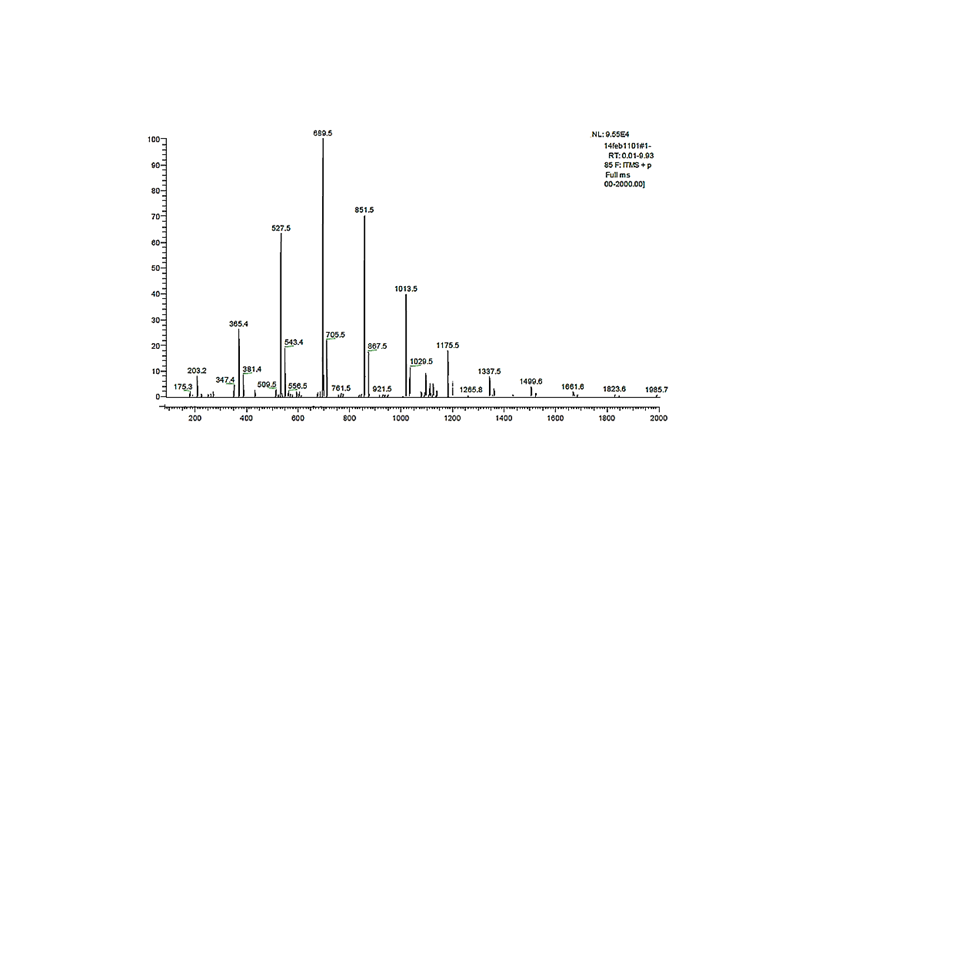

Supplement: S3 Fig — (TIF) [file pone.0179813.s003.tif]

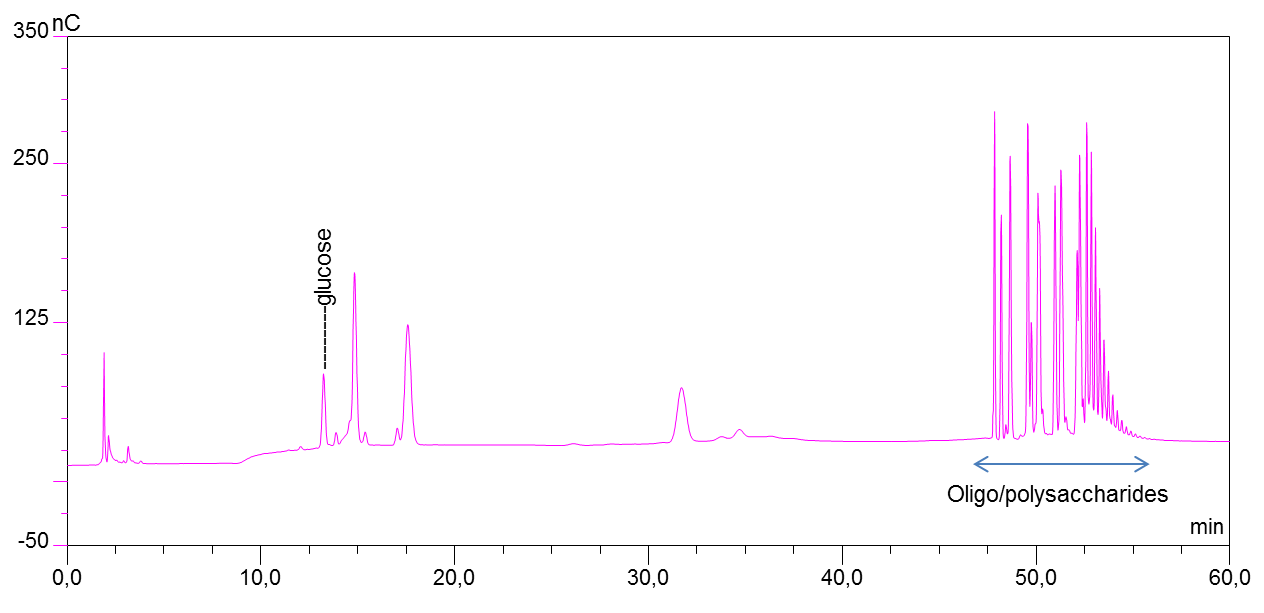

Supplement: S4 Fig — (TIF) [file pone.0179813.s004.tif]

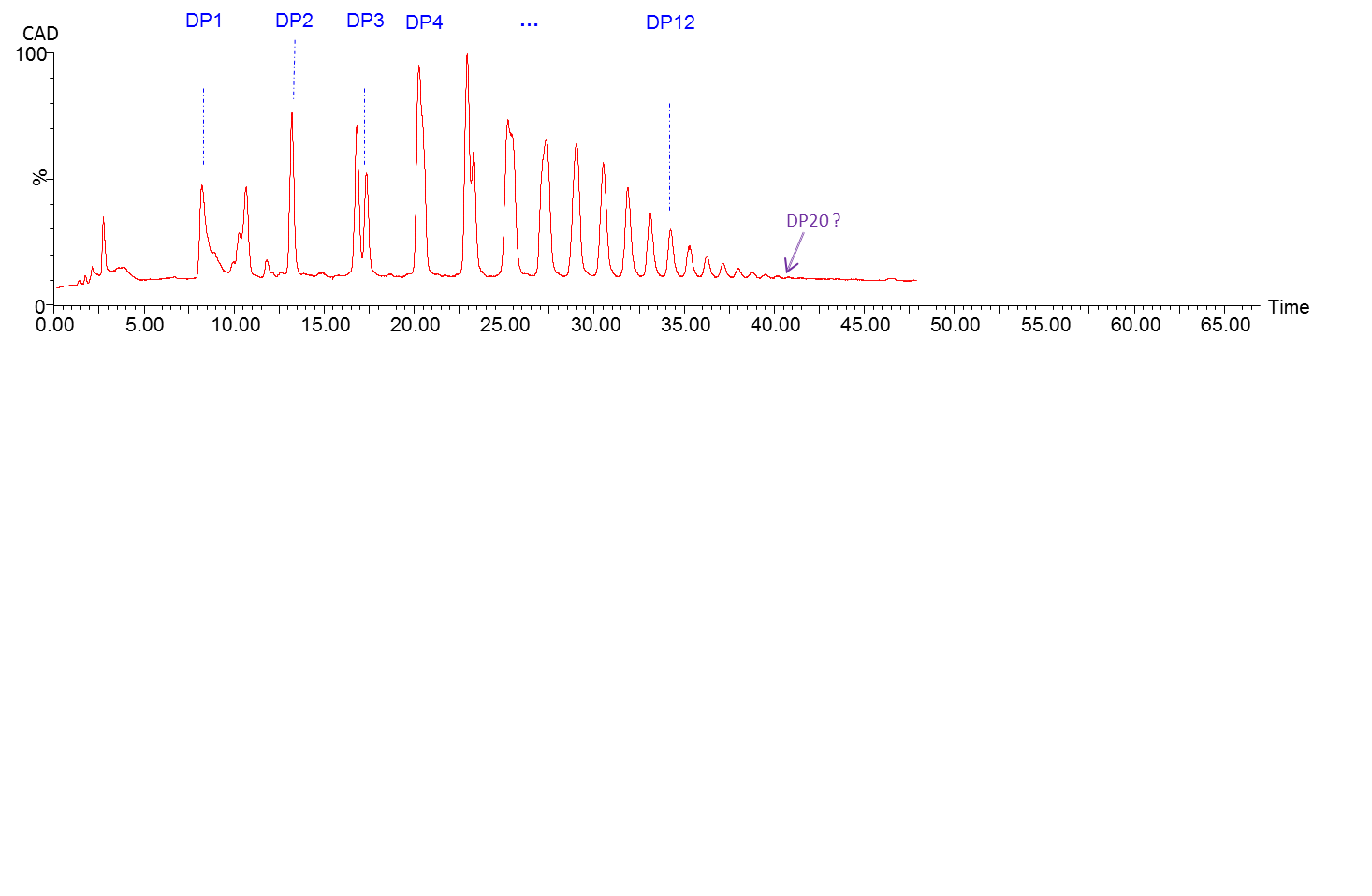

Supplement: S5 Fig — (TIF) [file pone.0179813.s005.tif]

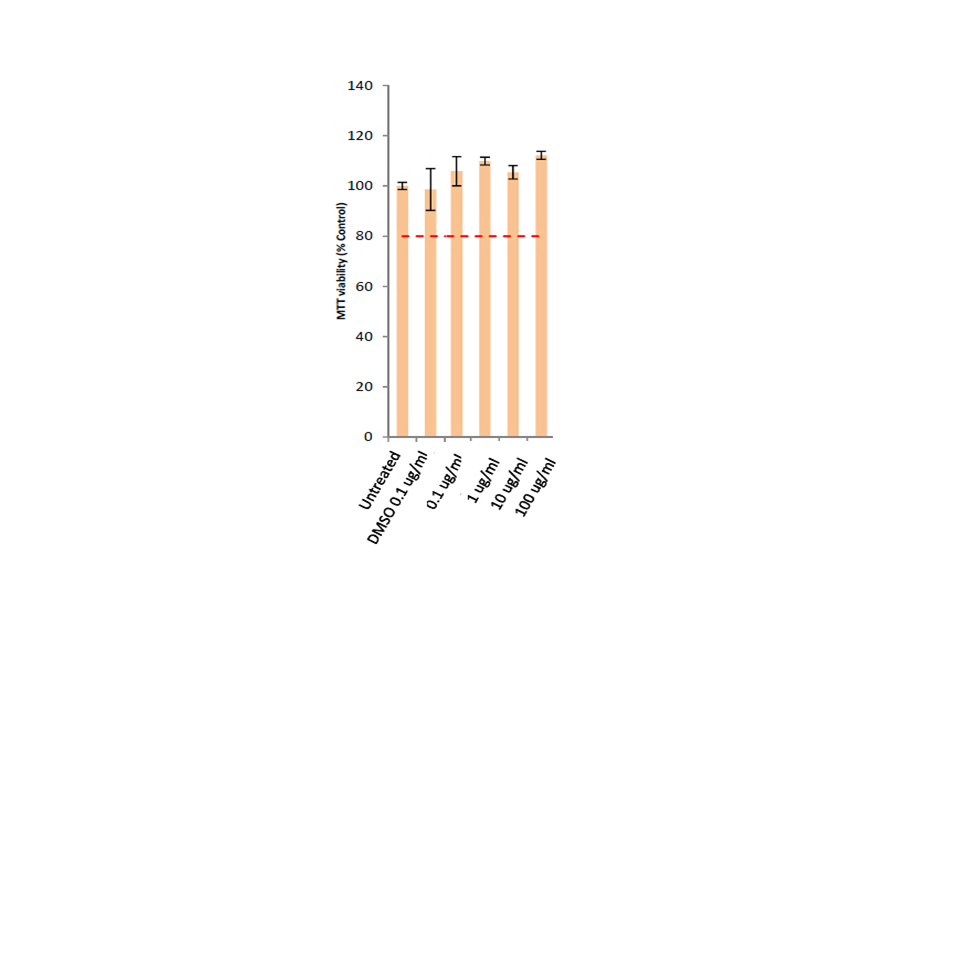

Supplement: S6 Fig — (TIF) [file pone.0179813.s006.tif]
